# Case Report: Digital CTA-assisted anterolateral thigh perforator pedicled flap for repairing postoperative defects in Vulvar Paget's disease

**DOI:** 10.3389/fmed.2026.1835423

**Published:** 2026-06-19

**Authors:** Yawen Bai, Yifan He, Ping Tang, Zesheng Zeng, Wei Wang, Haoming Luo, Jianfeng Sheng

**Affiliations:** Department of Thyroid, Head, Neck and Maxillofacial Surgery, The Third Hospital of Mianyang, Sichuan Mental Health Center, Mianyang, Sichuan, China

**Keywords:** anterolateral thigh (ALT) flap, case report, Computed tomographic angiography (CTA), digitalization, Vulvar Paget's disease (VPD)

## Abstract

**Objective:**

Vulvar Paget's disease (VPD) often requires extensive resection that may result in perineal tissue defects. The anterolateral thigh (ALT) flap is a commonly used reconstructive option; however, anatomical variations in its perforator vessels can pose surgical challenges. This study aims to evaluate the role of preoperative digital computed tomographic angiography (CTA) in ALT flap planning and to report our experience with vulvar reconstruction following Vulvar Paget's disease excision.

**Methods:**

Clinical data from a patient with Vulvar Paget's disease were retrospectively analyzed. Following wide local excision, vulvar reconstruction using an ALT flap was planned. Preoperative CTA combined with three-dimensional (3D) digital reconstruction technology was performed to localize and assess the perforator vessels of the anterolateral thigh, guiding the design of a customized flap. The flap was harvested and transferred according to the preoperative plan to reconstruct the vulvar defect.

**Results:**

Digital CTA successfully delineated the origin, course, and caliber of the perforator vessels, facilitating safe and precise flap harvest. Postoperatively, the flap demonstrated good perfusion, with satisfactory vulvar morphology and functional recovery. The donor site healed uneventfully.

**Conclusion:**

Preoperative CTA effectively assesses the perforator anatomy of the ALT flap, enhancing surgical predictability and safety. It serves as a valuable adjunct in vulvar reconstruction following Vulvar Paget's disease excision.

## 1 Introduction

Vulvar Paget's disease (VPD), a subtype of extramammary Paget's disease, constitutes approximately 1% of anogenital malignancies (1). It typically presents as slowly progressive infiltrative erythematous plaques with erosions and scaling in the perineal region and is frequently misdiagnosed. Surgical excision is the primary treatment; however, wide resection often results in significant tissue defects, posing major reconstructive challenges. Since its introduction in 1984 (2), the anterolateral thigh perforator flap has been widely adopted due to its long vascular pedicle, reliable blood supply, large harvestable area, favorable outcomes, and low complication rates. Although commonly used for trunk and extremity reconstruction, it is also effective for perineal repair (3).

The variability of perforator vessels, however, introduces surgical unpredictability. Precise preoperative visualization of perforator anatomy is therefore critical for successful surgery. Conventional two-dimensional (2D) imaging often struggles to intuitively display 3D vascular structures. Digital computed tomography angiography provides detailed three-dimensional mapping of perforators, enabling individualized and precise flap design (4).

Currently, reports on CTA-guided anterolateral thigh flap reconstruction for Vulvar Paget's disease defects remain scarce. This article presents a case of extensive Vulvar Paget's disease defect reconstructed with an anterolateral thigh flap under digital CTA guidance.

## 2 Clinical data

**Figure 1:**
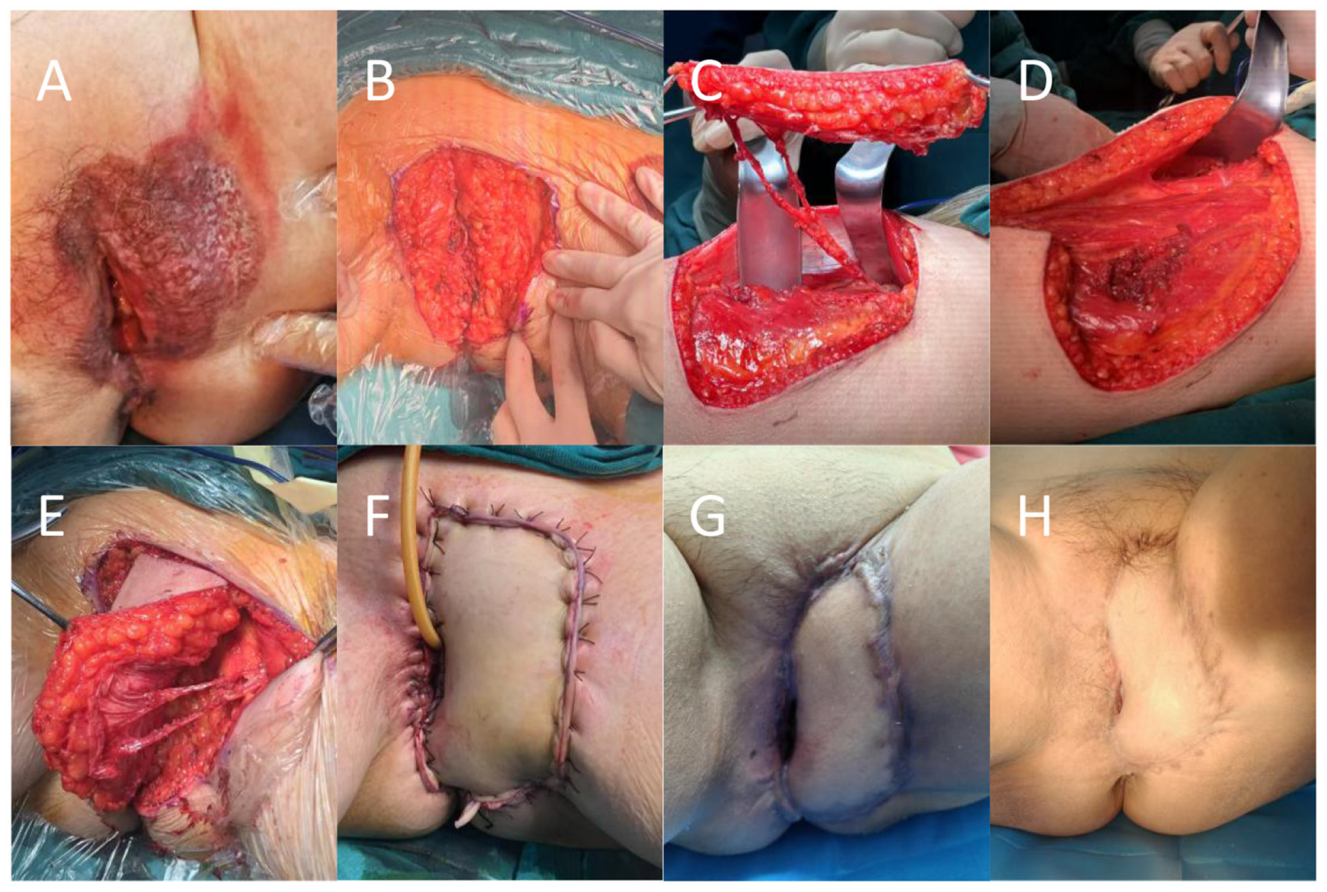
ALT flap reconstruction for Vulvar Paget's disease. **A**, preoperative Lesion Mapping. **B**, surgical Defect after Radical Vulvectomy. **C**, harvesting of the ALT Flap and Dissection of the Vascular Pedicle. **D**, creation of the Submuscular Tunnel through the Rectus Femoris for Flap Transposition. **E**, transposition of the Flap to the Vulvar Defect. **F**, final Reconstruction of Vulvar Contour and Donor-site Closure. **G**, 45 days after reconstruction. **H**, 1 year after reconstruction.

A 68-year-old woman patient presented with a 10-year history of vulvar pruritus that had progressively worsened over the past year, accompanied by vulvar lesions observed for more than 20 days. A prior biopsy performed at an external medical facility confirmed the diagnosis of Paget's disease, prompting a recommendation for surgical management. Clinical examination demonstrated erythematous, thickened lesions with scattered ulcerations involving both labia majora, extending to the thigh folds, as well as the perineal region and vaginal orifice. The left-sided lesion measured 10 × 7 cm, with extension to the left thigh fold, while the right-sided lesion measured 10 × 3 cm, involving the posterior fourchette and perianal skin (Figure 1A). Lymph node biopsy results confirmed the absence of metastatic involvement.

**Figure 2:**
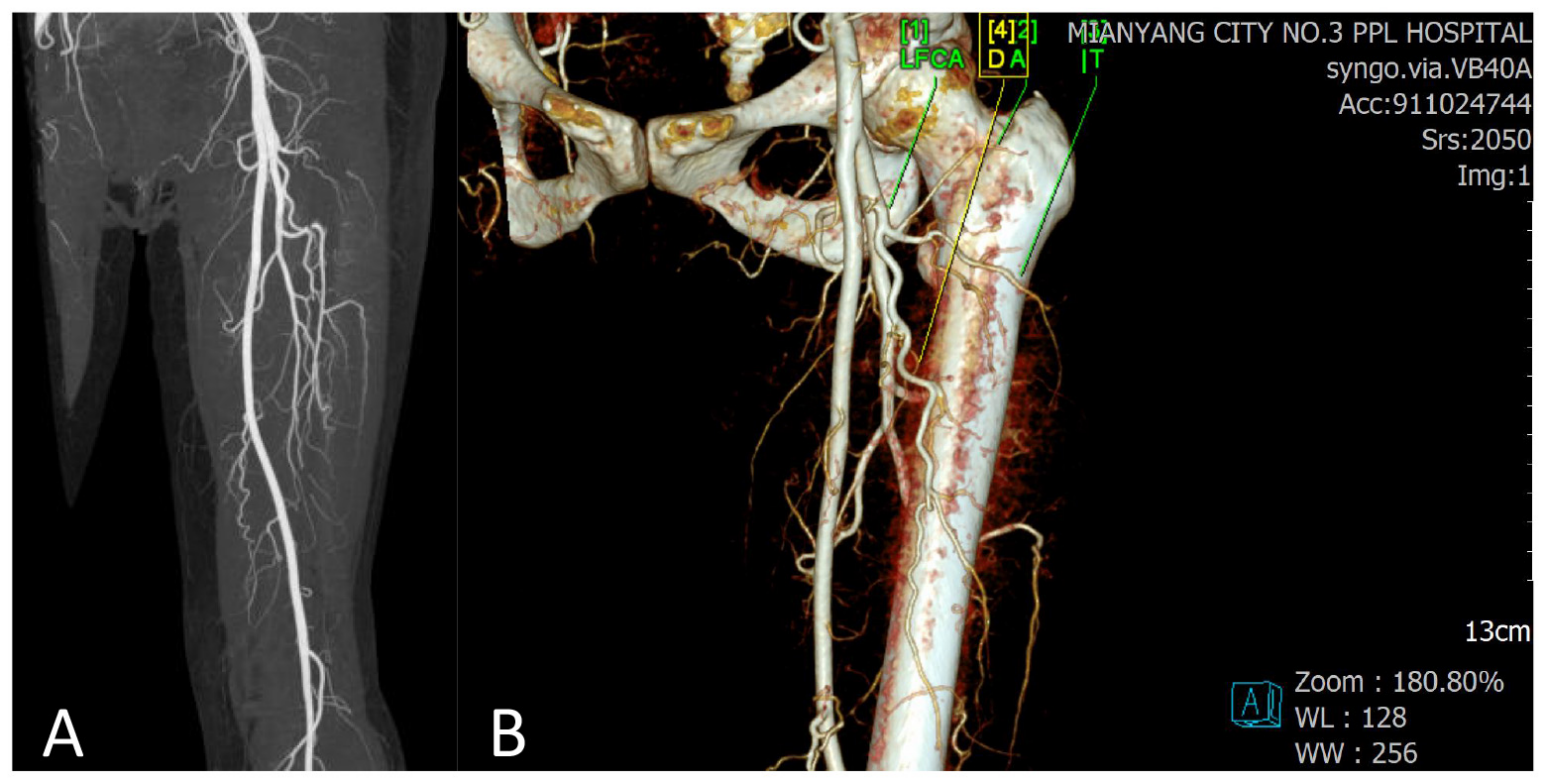
**A**, CTA image. **B**, Three-dimensional reconstruction of the perforator vessels of the anterolateral thigh flap.

**Figure 3:**
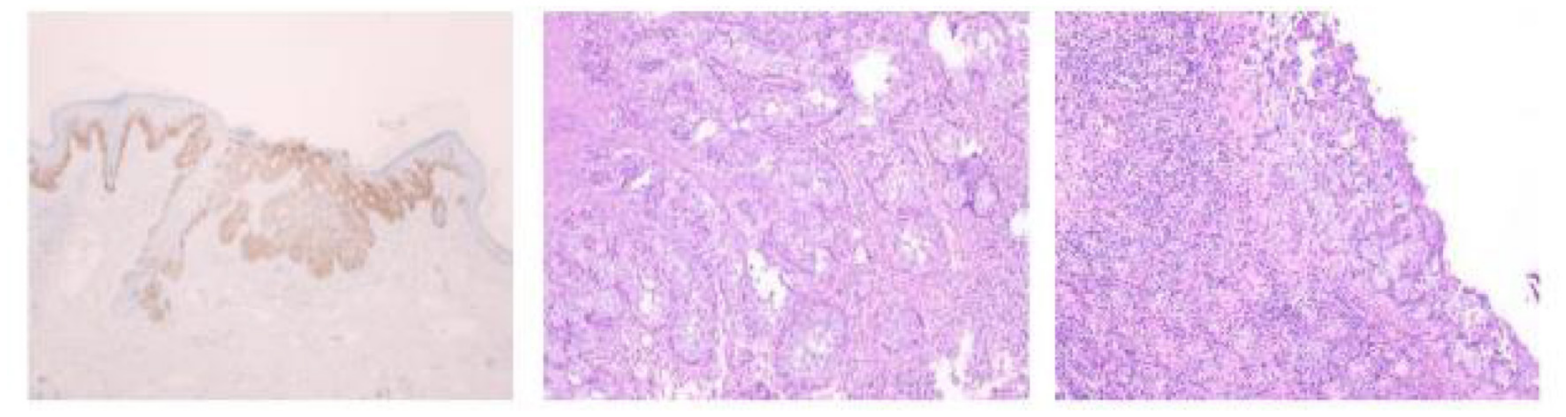
Pathological HE staining (×100), Postoperative pathological biopsy, immunohistochemical staining, and HPV genotyping diagnosis: Paget's disease, involving hair follicles, with small focal areas invading the superficial dermis. CEA(+), CK7(+), EMA(+), GCDFP-15 (focal positivity in minority of cells), HER2 (2+), P16(+), P40(-), S100(-), P53 (+, wild-type pattern, 10%), Ki-67 (+, 40%). High-risk HPV type: Type 51, positive.

Following comprehensive multidisciplinary assessment and meticulous preoperative evaluation, the patient was diagnosed with clinical stage IB vulvar Paget's disease (VPD), necessitating radical vulvectomy. In anticipation of the substantial surgical defect, reconstruction using a left anterolateral thigh (ALT) flap was planned. Preoperative 64-slice spiral computed tomography angiography (CTA) (Figure 2A) of the left lower limb vasculature was performed, utilizing maximum intensity projection (MIP) and volume rendering (VR) techniques. This enabled three-dimensional reconstruction of the ascending, transverse, and descending branches of the lateral circumflex femoral artery (Figure 2B). The imaging data not only confirmed the presence of a dominant perforator but also precisely measured its fascial emergence point and intramuscular trajectory. During the surgical procedure, the tumor was resected with 2-cm macroscopic margins (Figure 1B). Based on the localization provided by the preoperative digital CTA, the flap design was directly centered on the dominant musculocutaneous perforator of the descending branch. The ALT flap was harvested via a 12-cm transverse incision overlying the rectus femoris muscle, with careful preservation of perforating vessels. Because the perforator location was clarified in advance, this preoperative guidance directly altered the traditional harvesting strategy, completely avoiding extensive subfascial blind dissection and exploration, maximally preserving the surrounding normal vascular networks, and potentially reducing perforator separation time. The vascular pedicle was meticulously dissected to achieve sufficient length (Figure 1C), and the flap was subsequently transposed submuscularly through the rectus femoris tunnel to the vulvar defect (Figures 1D, E). Primary closure was achieved for the right-sided defect, while the vulva was successfully reconstructed (Figure 1F), with particular attention given to preserving the lateral femoral cutaneous nerve. Histopathological examination confirmed the diagnosis of Paget's disease (Figure 3).

The patient was administered antibiotics, anticoagulation therapy, and microcirculation management without any adverse events. Following discharge on postoperative day 15, clinical evaluations at 45 days (Figure 1G) and 1 year (Figure 1H) demonstrated well-perfused flaps, complete wound healing, and absence of disease recurrence. Regarding functional recovery, patient satisfaction was high: urinary function was normal; sitting tolerance was excellent without traction pain; partial protective sensory recovery was achieved in the flap's periphery; and there was no severe scar contracture restricting the vaginal orifice.

## 3 Discussion

Vulvar-perineal defect (VPD) reconstruction following radical excision presents a complex clinical challenge requiring multidisciplinary collaboration. This investigation demonstrates the efficacy of digital computed tomography angiography (CTA)-guided anterolateral thigh (ALT) flap reconstruction, which provides three distinct clinical benefits: optimal anatomical congruence, preoperative digital surgical planning, and gender-appropriate design considerations.

For this patient's extensive, irregular bilateral defects (left 10 × 7 cm; right 10 × 3 cm with perianal extension), the pedicled ALT flap offered distinct advantages over conventional options: local labial flaps lacked sufficient tissue volume, vertical rectus abdominis myocutaneous (VRAM) flaps carried a 16.7% abdominal hernia risk (5), and superficial circumflex iliac artery perforator (SCIP) flaps had a shorter vascular pedicle that restricted tension-free coverage of the posterior perineum (Table 1, Supplementary Digital Content 1. A table that shows comparative analysis of commonly used flaps in female vulvar reconstruction). In contrast, the ALT flap provides three distinct advantages: 1. consistent perforator localization within 1 cm of the midpoint along the anteroposterior (AP) line, facilitating tension-free transposition (6); 2. maintenance of native vascular supply, thereby reducing operative duration relative to free flap alternatives; and 3. favorable sensory recovery outcomes (demonstrating 0.5 cm two-point discrimination at 12 months postoperatively, achieving S3+ sensory grade) (7).

**Table 1:** Comparison of commonly used flaps for vulvar reconstruction.

Flap type	ALT	TRAM	DIEP	SCIP
Muscle function preservation	√	×	√	√
Pedicle length and arc of rotation	√ Long, covers entire perineum	√ Long	√ Moderate	× Short, limited rotation
Potential for sensory nerve preservation	√ Possible (sensory nerves)	×	×	√ Possible
Aesthetic Softness and natural appearance	√ Excellent, moderate skin and fat	× Bulky	√ Abundant fat	× Thin fat layer
Donor site morbidity	√ Minimal, well-concealed	× Abdominal wall weakness risk	√ Requires abdominal dissection	√ Small incision

ALT, Anterolateral Thigh perforator flap; TRAM, Transverse Rectus Abdominis Myocutaneous flap; DIEP, Deep Inferior Epigastric Perforator flap; SCIP, Superficial Circumflex Iliac Artery Perforator flap.

Preoperative digital computed tomographic angiography (CTA) effectively addresses perforator variability. In contrast to Doppler ultrasonography which often lacks precision, 64-slice CTA with maximum intensity projection (MIP) and volume rendering (VR) techniques enables clear, stereoscopic mapping of the dominant perforator location, caliber, and intramuscular trajectory (12%−40% septocutaneous vs. 60%−88% musculocutaneous variants) (8). In actual practice, this digital guidance allowed us to abandon time-consuming full vascular exploration and directly locate the incision based on the virtual 3D images, thereby potentially minimizing intraoperative exploration time and mitigating vascular compromise risks. This approach proves particularly advantageous in obese patients (BMI > 28).

Gender-specific refinements in surgical technique optimized both aesthetic and functional outcomes: (1) A “chimeric” flap design incorporating layered fascial and adipose tissue transplantation effectively replicated labial volume (9); (2) Preservation of a 5 cm proximal epidermal segment facilitated the creation of a naturalistic, irregular mucosal transition, reducing postoperative contracture. One-year follow-up assessments confirmed satisfactory functional and cosmetic results among female recipients.

Study limitations include its single-center design, absence of standardized perforator selection criteria for multi-perforator flap configurations, and insufficient consideration of age-dependent vascular alterations. Future multicenter investigations should prioritize the development of CTA-based perforator grading systems and patient-specific 3D-printed surgical guides to transition from anatomical reconstruction to comprehensive functional restoration.

## 4 Conclusion

The pedicled anterolateral thigh (ALT) flap represents a robust and dependable technique for vulvar reconstruction following vulvar and perineal defect (VPD) excision. Preoperative digital computed tomography angiography (CTA)-guided spatial 3D navigation may facilitate the localization of the dominant perforator and flap design, potentially mitigating the risks of blind intraoperative exploration and optimizing surgical efficiency. Continued advancements in microsurgical techniques and digital preoperative planning suggest expanding clinical utility for this approach in complex vulvar reconstruction, with demonstrable benefits for long-term patient outcomes and quality of life.

## Data Availability

The original contributions presented in the study are included in the article/supplementary material, further inquiries can be directed to the corresponding author.
